# Supplementary material for: SIR-SI model with a Gaussian transmission rate: Understanding the dynamics of dengue outbreaks in Lima, Peru
Source: PLoS One. 2023 Apr 13;18(4):e0284263. doi: 10.1371/journal.pone.0284263 (PMC10101463; doi:10.1371/journal.pone.0284263)
Supplement: S1 File — (PDF) [file pone.0284263.s001.pdf]

Supporting information

S1 Appendix. Temperature Dependent Parameters

- The biting rate b is given by

$$b(T) = \begin{cases} 0.000202T(T-13.35)\sqrt{40.08-T} & \text{for } 13.35°C \leq T \leq 40.08°C, \\ 0 & \text{for } T < 13.35°C, 40.08°C < T, \end{cases} \tag{20}$$

- The probability b_h of infection from mosquito to human per bite is given by

$$b_h(T) = \begin{cases} 0.001044T(T-12.286)\sqrt{32.461-T} & \text{for } 12.286°C \leq T \leq 32.461°C, \\ 0 & \text{for } T < 12.286°C, 32.461°C < T, \end{cases} \tag{21}$$

- The probability b_v of infection from human to mosquito per bite is given by

$$b_v(T) = \begin{cases} -0.9037 + 0.0729T & \text{for } 12.4°C \leq T \leq 26.1°C, \\ 1 & \text{for } 26.1°C \leq T \leq 32.5°C, \\ 0 & \text{for } T < 12.4°C, 32.5°C < T. \end{cases} \tag{22}$$

- The mortality rate μ_v of an adult mosquito is given by

$$\mu(T) = 8.692 \cdot 10^{-1} - 1.590 \cdot 10^{-1}T + 1.116 \cdot 10^{-2}T^2 - 3.408 \cdot 10^{-4}T^3 + 3.809 \cdot 10^{-6}T^4. \tag{23}$$

S2 Appendix. Analysis of the model We analyzed the disease-free equilibrium point with exogenous and temperature variable terms. The basic reproduction number R_0 was computed by using the next-generation matrix evaluated at the disease-free equilibrium point [1-3]. At this equilibrium point, the vector population (i.e., mosquitoes) tends to decrease because of the environmental conditions, natural lifespan, and artificial external conditions such as insecticides [4] or control methods [4]. In addition, we considered the progression of cases by using the effective reproduction number R_t.

Proposition 1. *Consider the SIR-SI model given by (2)–(6). For the host-to-vector transmission rate $\beta_{hv} = \beta_{ex}\, b\, b_h$, and vector-to-human transmission rate $\beta_{vh} = \beta_{ex}\, b\, b_v, \beta_{ex}$ is defined by (11). The biting rate $b(T)$ is defined by (20), the probability of mosquito to human infection per bite $b_h(T)$ is given by (21), and the probability of human to mosquito infection per bite $b_v(T)$ is given by (22). Then, the basic reproduction number R_0 is given by*

$$R_0 = \beta_{ex}\, \frac{b(T)}{N_h} \left| \sqrt{\frac{b_h(T) S_v}{\gamma} \frac{b_v(T) S_h}{\mu}} \right|. \tag{24}$$

Proof. First, (2)–(6) are reduced to

$$\frac{dI_h}{dt} = \beta_{vh} \frac{S_h}{N_h} I_v - \gamma I_h \tag{25}$$

$$\frac{dI_v}{dt} = \beta_{hv} S_v \frac{I_h}{N_h} - \mu I_v. \tag{26}$$

Linearizing the above equations and evaluating the linear form at the disease-free equilibrium point $E_{df} = (S_h, I_h, R_h, S_v, I_v) = (N_h, 0, 0, N_v, 0)$ obtains

$$\dot{\xi} = J\xi \tag{27}$$

where the matrix J is the Jacobian associated with (25) evaluated at E_{df}. By simplicity, the matrix J is decomposed into $J = F - V$:

$$J = F - V = \begin{bmatrix} 0 & \beta_{vh} \frac{S_h}{N_h} \\ \beta_{hv} \frac{S_v}{N_h} & 0 \end{bmatrix} - \begin{bmatrix} \gamma & 0 \\ 0 & \mu \end{bmatrix}.$$

The dynamics of (27) can be determined by the eigenvalues of the matrix J. The matrix $M = FV^{-1}$ is of particular interest and is given by [2]

$$M = \left[\begin{array}{cc} 0 & \beta_{vh} \frac{S_h}{N_h} / \mu \\ \beta_{hv} \frac{S_v}{N_h} / \gamma & 0 \end{array} \right].$$

The reproduction number R_0 is obtained from the spectral radius of M:

$$\rho(M) = \left| \sqrt{\frac{\beta_{vh}\, S_v}{\gamma N_h} \frac{\beta_{hv}\, S_h}{\mu N_h}} \right|.$$

For $\beta_{hv} = \beta_{ex}\, b\, b_h$ and $\beta_{vh} = \beta_{ex}\, b\, b_v$ and the corresponding functions for β_{ex}, $b(T)$, $b_h(T)$, and $b_v(T)$, the following is obtained:

$$R_0 = \beta_{ex}\, b(T) \left| \sqrt{\frac{b_h(T)\, S_v}{\gamma N_h} \frac{b_v(T)\, S_h}{\mu(T) N_h}} \right|. \tag{28}$$

Remark 1. *Since we were interested in when R_0 is comparable to 1, we can use*

$$R_0 = \beta_{ex}^2\, b^2(T) \frac{b_h(T)\, S_v}{\gamma N_h} \frac{b_v(T)\, S_h}{\mu(T) N_h}.$$

It is possible to observe that the transition is at $R_0 = 1$. The disease-free equilibrium point is asymptotically stable if and only if $R_0 < 1$, and the epidemic is settled if $R_0 > 1$.

In the case of Lima, the situation was strongly affected by external conditions including the environmental, inflow cases due to travelers (which were not considered in this model), and entomological conditions. An exogenous parameter was introduced to model external forces that affect the dynamics of the outbreak.

The epidemiological curves of the SIR-SI model (2) including the transmission rate (9) and exogenous parameter (11) were fitted to observed data by adjusting the parameters u, σ, and k (related to the β_{ex}). We used a maximum likelihood function as a cost function in the optimization solver. We used a normal distribution function for the probability under the assumption that the observation error had a normal distribution.

Corollary 1. *If the functions and values for b, b_h, b_v, β_{ex}, $N_v = 2N_h$, $S_h = 1$, and $S_v = 2S_h$ are given, then R_0 (24) is bounded by*

$$0 \leq R_0 \leq k \sqrt{\frac{1}{\gamma \mu(T)}} \tag{29}$$

Proof. The proof of this corollary is obtained directly from the definitions of b, b_h, b_v, and β_{ex}.

The basic reproduction number (24) measures the epidemic potential of a pathogen, which in this case is dengue [2]. It is defined as the average number of new infections created by an infectious individual in an entirely susceptible population. In practice, the situation evolves, so the data are used to estimate the effective reproduction number R_t. The quantitative changes in the effective reproduction number need to be monitored to determine when it approaches the critical threshold $R_t = 1$ [5]. In the case of Lima, the reports of few cases would imply a small R_0. However, as the outbreak evolves, R_t can increase and even exceed the critical value of $R_t = 1$.

S3 Appendix. Case progression for an infected case Here, we consider the temperature-dependent SIR-SI model with an exogenous variable as expressed in (2). The total number of cases up to time t is represented by $Q(t)$ and is calculated as follows:

$$\frac{dQ}{dt} = \beta_{vh}\frac{S_h}{N_h}I_v \tag{30}$$

To consider the evolution of R_t, we followed the approach of Bettencourt and Ribeiro [5]. We considered that epidemic reports generally state the occurrence of new infected cases within the period τ. Hence, the total number of cases is given by $Q(t+\tau) - Q(t) = \Delta Q(t+\tau)$.

The change in cases between t and $t+\tau$ is obtained by

$$I_h(t+\tau) = I_h(t)\text{exp}\left(\int_t^{t+\tau}\left(-\gamma + \beta_{vh}\frac{S_h(t)}{N_h(t)}p\right)dt\right) \tag{31}$$

$$I_v(t+\tau) = I_v(t)\text{exp}\left(\int_t^{t+\tau}\left(-\mu + \beta_{hv}\frac{S_v(t)}{N_h(t)}\frac{1}{p}\right)dt\right) \tag{32}$$

where $p = \frac{I_v}{I_h}$. For theoretical purposes, we can consider the number of mosquitoes to remain constant from the time t to $t+\tau$. Hence, $I_v(t+\tau) = I_v(t)$. Thus, in (32) the value of p must satisfy

$$p = \frac{\beta_{hv}S_v(t)}{\mu N_h(t)}. \tag{33}$$

Introducing (33) to (31) obtains

$$I_h(t+\tau) = I_h(t)\text{exp}\left(\gamma\int_t^{t+\tau}\left(-1 + \frac{\beta_{vh}}{\gamma}\frac{S_h(t)}{N_h(t)}\frac{\beta_{hv}}{\mu}\frac{S_v(t)}{N_h(t)}\right)dt\right), \tag{34}$$

where the effective reproduction number $R_t(t)$ can be calculated directly as

$$R_t(t) = \frac{S_h(t)}{N_h(t)}R_0.$$

The number of infectious cases decreases if $R_t < 1$ or, equivalently,

$$\left(-1 + \frac{\beta_{vh}}{\gamma}\frac{S_h(t)}{N_h(t)}\frac{\beta_{hv}}{\mu}\frac{S_v(t)}{N_h(t)}\right) < 0.$$

In general, the assumption that $I_v(t)$ remains constant during the evaluation period is adequate for emerging infectious diseases (i.e., few cases within a much larger population), which is the case for Lima. To compute R_t, we used Bettencourt and Ribeiro's [5] algorithm.

References

[1] Lee H, Kim JE, Lee S, Lee CH. Potential effects of climate change on dengue transmission dynamics in Korea. PLoS One. 2018;13(6):e0199205.

[2] Brauer F, Castillo-Chavez C. Mathematical Models for Communicable Diseases. Philadelphia, PA: Society for Industrial and Applied Mathematics; 2012. Available from: https://epubs.siam.org/doi/abs/10.1137/1.9781611972429.

[3] Kim JE, Lee H, Lee CH, Lee S. Assessment of optimal strategies in a two-patch dengue transmission model with seasonality. PloS one. 2017;12(3):e0173673.

[4] Pérez-Estigarribia PE, Bliman PA, Schaerer CE. A class of fast-slow models for adaptive resistance evolution. Theoretical Population Biology. 2020;135:32–48.

[5] Bettencourt LMA, Ribeiro RM. Real Time Bayesian Estimation of the Epidemic Potential of Emerging Infectious Diseases. PLOS ONE. 2008;3(5):1–9. doi:10.1371/journal.pone.0002185.
